# Supplementary material for: Effects of Flupyradifurone and Two Reference Insecticides Commonly Used in Toxicological Studies on the Larval Proteome of the Honey bee Apis mellifera
Source: Insects. 2023 Jan 12;14(1):77. doi: 10.3390/insects14010077 (PMC9862931; doi:10.3390/insects14010077)
Supplement: Supplementary file 1 [file insects-14-00077-s001.zip › S1-Supplementary data peptides.pdf]

Supplementary Materials S1

Effects of flupyradifurone and two reference insecticides, commonly used in toxicological studies, on the larval proteome of the honey bee Apis mellifera

Arne Kablau[1,2], Silvio Erler[3,4], Jakob H. Eckert[3,5], Jens Pistorius[3], Soroush Sharbati[1] and Ralf Einspanier[1*]

1 Institute of Veterinary Biochemistry, Freie Universität Berlin, Germany
2 LABOKLIN GmbH and Co. KG, Bad Kissingen, Germany
3 Institute for Bee Protection, Julius Kühn Institute (JKI) - Federal Research Centre for Cultivated Plants, Braunschweig, Germany
4 Zoological Institute, Technische Universität Braunschweig, Germany
5 Institute for Microbiology, Technische Universität Braunschweig, Germany
* Correspondence: Ralf.Einspanier@fu-berlin.de; Tel.: +493083862575

S1-Supplementary peptide data

Matched peptides of five proteins after 2D-gelelectrophoresis and identification via LC/MS.

1 - Matched peptides for **3-Ketoacyl-CoA thiolase** are shown in ***bold red***.

1 MSLVTK**GIFI VAAKRTPFGS FGGK**FMNK**NA TDLSVIAAK**S ALQSAKLNPE

51 KVDNVIFGHV LPISSSDGGF LTRHVALK**SG IPLEKPSFSL NR**LCGSGFQS

101 IVSGAQSILL GESKIVLTGG VENMSQVPFS VRNIRFGTSL GQKYQFEDSL

151 WLGFSDTYCN LSMGETAEK**L GSQYNLKRQE VDEFALR**SQQ LWKAANDAGR

201 **FNEEIAPVTI TVKKQDIVVN TDEHPRPQTT LQNLSKLPSI FK**KDGLVTAG

251 SSSGISDGAG VIILASEEVI K**TEQLTPLAR** LVGYSVVGVE PSIMGIGPVP

301 AIQKLLKITN KTLNDIELIE INEAFGSQTL ACVKELKLDI NK**LNVDGGSI**

351 **ALGHPLAASG SRITAHLVHE LR**RRKTGSLG IGSACIGGGQ GIALMIEAL

2 - Matched peptides for m**ajor royal jelly protein 2** are shown in ***bold red***.

1 MTRWLFMVAC LGIACQGAIV RENSPRNLEK SLNVIHEWKY FDYDFGSEER

51 RQAAIQSGEY DHTKNYPFDV DQWRDK**TFVT ILRYDGVPST LNVISGK**TGK

101 GGRLLKPYPD WSFAEFKDCS KIVSAFK**IAI DKFDR**LWVLD SGLVNRTVPV

151 CAPK**LHVFDL K**TSNHLKQIE IPHDIAVNAT TGKGGLVSLA VQAIDLANTL

201 VYMADHK**GDA LIVYQNADDS FHRLTSNTFD YDPR**YAK**MTI DGESFTLK**NG

251 ICGMALSPVT NNLYYSPLAS HGLYYVNTAP FMK**SQFGENN VQYQGSEDIL**

301 **NTQSLAK**AVS KNGVLFVGLV GNSAVGCWNE HQSLQR**QNLE MVAQNDRTLQ**

351 **MIAGMK**IKEE LPHFVGSNKP VKDEYMLVLS NRMQKIVNDD FNFDDVNFRI

401 LGANVKELIR NTHCVNNNQN DNIQNTNNQN DNNQKNNKKN ANNQKNNNQN

451 DN

3 - Matched peptides for **glutathione S-transferase S1-like** are shown in ***bold red***.

1 MTERQPLYKL IYFNARGRAE HIRYIFAYAG IDYVDERILK ECWPELKKSM

51 PYGMLPVLEI NGKPIAQSNA VARYLARKHN LTGRDEWEAM MCDVLVDTLG

101 DLKQFISQYR IEEDLFKKKE KKMKLLKETI PFYLNKFEQI ISENGSYTVG

151 TTTTWADFVF AVALENFENI FGTMALENYP GLRALKKRVH EIPSIVNWLS

201 KRPHTEFGRV LLLFHFTNLI RNFYLYSKMS TYK**LTYFPVK GLGEPIRFLL**

251 SYAGISFEDE RFDRDDWPKI KPTTPFGQVP VLDVDGKKIA QSVAISRYLA

301 KKSGLAGKDD WEALEIDSIV DTIHDVRARL **AAFHYEENEE IKAAK**R**KIAD**

351 EVVPYYLERL DAQVKNNGGY FVGGALSWAD LSFVALLDYL NFMNGSDLIE

401 K**YDNLKQLKE KVLNLPAIKS WLDKRPHSDF**

4 - Matched peptides for **retinal dehydrogenase 1** are shown in ***bold red***.

1 MKMNRKIDIK **YTQLFINNNF VDSVSGKKFS TINPADGSVI AEISEGDKAD**

51 VDKAVAAAKE AFSRKSKWRT MDASVRGNLM NK**FADLIARD LEYIATLETL**

101 DNGKTYSDAL FDIEASIQTI RYYASWCDKI FGDTIPADGN LISLTRKEPV

151 GVVGQIIPWN YPFLMLAWK**W SPALATGCTI VLKPAEQTPL SALYAAALAK**

201 EAGFPPGVIN VITGYGPTAG AAIAEHPEIN KIAFTGSTEV GHLIMAASAK

251 SNLK**RVSLEL GGKSPLVIFD DVDIKEAAEI AFNAIFANHG QNCCAGSR**TF

301 VHAK**IYDQFV NHAK**QLASQR **KVGDPFNSET QQGPQIDEEM FHK**VLSLIK**S**

351 GQEEGAVLET GGERHGNVGY FIKPTVFSNV TDTMRIAKEE IFGPVQSILK

401 FETMDEVIER ANNTNYGLAA GVVTKNIDKA LIFAQAVEAG SVWINCYDAI

451 TPQTPFGGFK QSGIGR**ELGA EGLKEYLETK** TISIKVSNN

5 - Matched peptides for **14-3-3 protein zeta** shown in ***bold red***.

1 MSVDKEELVQ RAK**LAEQAER YDDMAAAMKA VTETGVELSN EERNLLSVAY**

51 KNVVGARRSS WR**VISSIEQK TEGSER**KQQM AKEYREKVEK ELR**EICYDVL**

101 GLLDKYLIPK ASNAESKVFY LKMKGDYYR**Y LAEVATGETR NAVVDDSQKA**

151 YQDAFEISKS K**MQPTHPIR**L GLALNFSVFY YEILNSPDKA CQLAK**QAFDD**

201 AIAELDTLNE DSYKDSTLIM QLLRDNLTLW TSDTQGDADE AQEGGDN
