# Supplementary material for: Effects of Flupyradifurone and Two Reference Insecticides Commonly Used in Toxicological Studies on the Larval Proteome of the Honey bee Apis mellifera
Source: Insects. 2023 Jan 12;14(1):77. doi: 10.3390/insects14010077 (PMC9862931; doi:10.3390/insects14010077)
Supplement: Supplementary file 1 [file insects-14-00077-s001.zip › S2-Supplementary data peptides.pdf]

## Supplementary Materials S2

Effects of flupyradifurone and two reference insecticides, commonly used in toxicological studies, on the larval proteome of the honey bee Apis mellifera

Arne Kablau[1,2], Silvio Erler[3,4], Jakob H. Eckert[3,5], Jens Pistorius[3], Soroush Sharbati[1] and Ralf Einspanier[1*]

1 Institute of Veterinary Biochemistry, Freie Universität Berlin, Germany
2 LABOKLIN GmbH and Co. KG, Bad Kissingen, Germany
3 Institute for Bee Protection, Julius Kühn Institute (JKI) - Federal Research Centre for Cultivated Plants, Braunschweig, Germany
4 Zoological Institute, Technische Universität Braunschweig, Germany
5 Institute for Microbiology, Technische Universität Braunschweig, Germany
* Correspondence: Ralf.Einspanier@fu-berlin.de; Tel.: +493083862575

Description of LC/MS methodology used (provided by Proteome Factory, Berlin):

Protein identification was performed by Proteome Factory (Proteome Factory AG, Berlin, Germany). Protein bands were rebuffered by repeated shrinking (60% acetonitrile, 50 mM TEAB) and swelling steps (50 mM TEAB). For Cysteine alkylation, the swelling buffer first contained 10 mM TCEP, then 10 mM 2-iodoacetamide. In-gel proteolysis was done with sequencing grade porcine trypsin (Promega, Mannheim, Germany) overnight. For analysis, the samples were acidified with 2% formic acid. The nanoHPLC-ESI-MS/MS system consisted of an Dionex Ultimate 3000 nanoHPLC system (Thermo Scientific, Germering, Germany), nanoelectrospray emitter (Fossiliontech, Madrid, Spain) and an Orbitrap Velos mass spectrometer (Thermo Scientific, Bremen, Germany). Peptides were first trapped and desalted on the enrichment column (PepMap-C18, 0.3 x 5 mm, Dionex) for five minutes (solvent: 0.5% acetonitrile/0.5% formic acid), then separated on a ReproSil-Pur 120 C18-AQ column (0.075 x 500 mm column, Dr. Maisch, Ammerbuch-Entringen, Germany) using a linear gradient from 12% to 40% B (solvent A: water, solvent B: acetonitrile, both with 0.1% formic acid) with a total run time of 35 minutes. Ions of interest were data-dependently subjected to MS/MS according to the expected charge state distribution of peptide ions. Proteins were identified by database search against the Apis mellifera subset (23491 sequences) of the RefSeq protein database (National Center for Biotechnology Information, Bethesda, USA) and a contaminant database using MS/MS ion search of the Mascot search engine (Matrix Science, London, England). Only peptides matches with a score of 20 or above were accepted. Protein matches were required to have at least two significant unique sequences.
