# Supplementary material for: Effects of Flupyradifurone and Two Reference Insecticides Commonly Used in Toxicological Studies on the Larval Proteome of the Honey bee Apis mellifera
Source: Insects. 2023 Jan 12;14(1):77. doi: 10.3390/insects14010077 (PMC9862931; doi:10.3390/insects14010077)

S3. Supplementary data:
Gel images of all replicates performed

Control group:

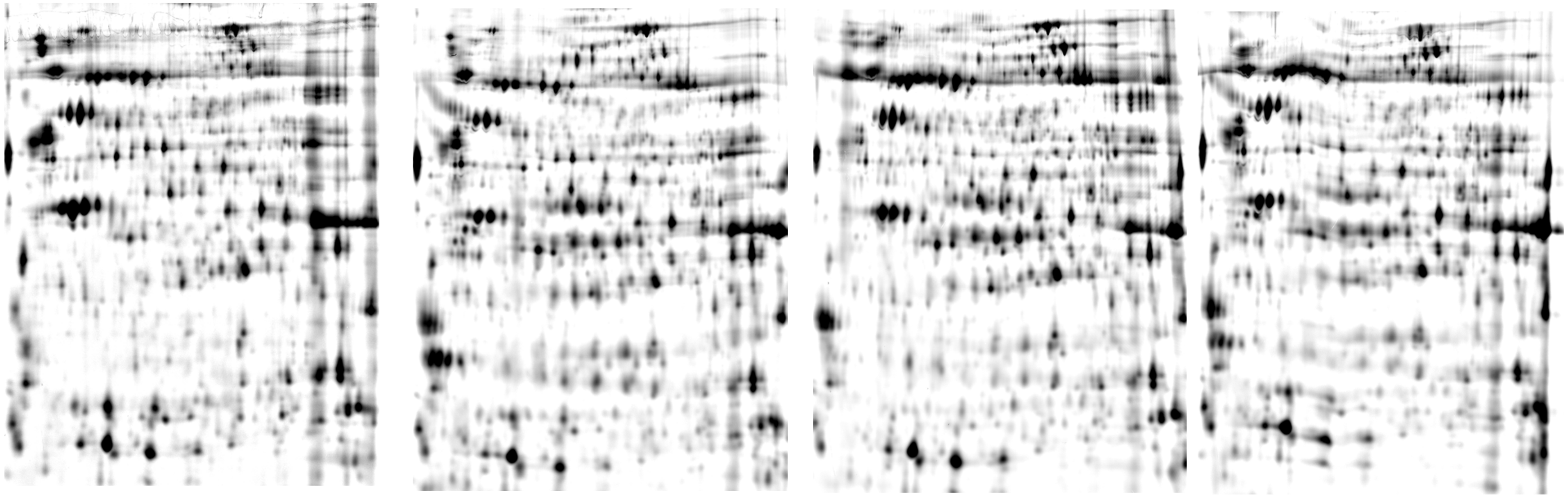

Control solvent group:

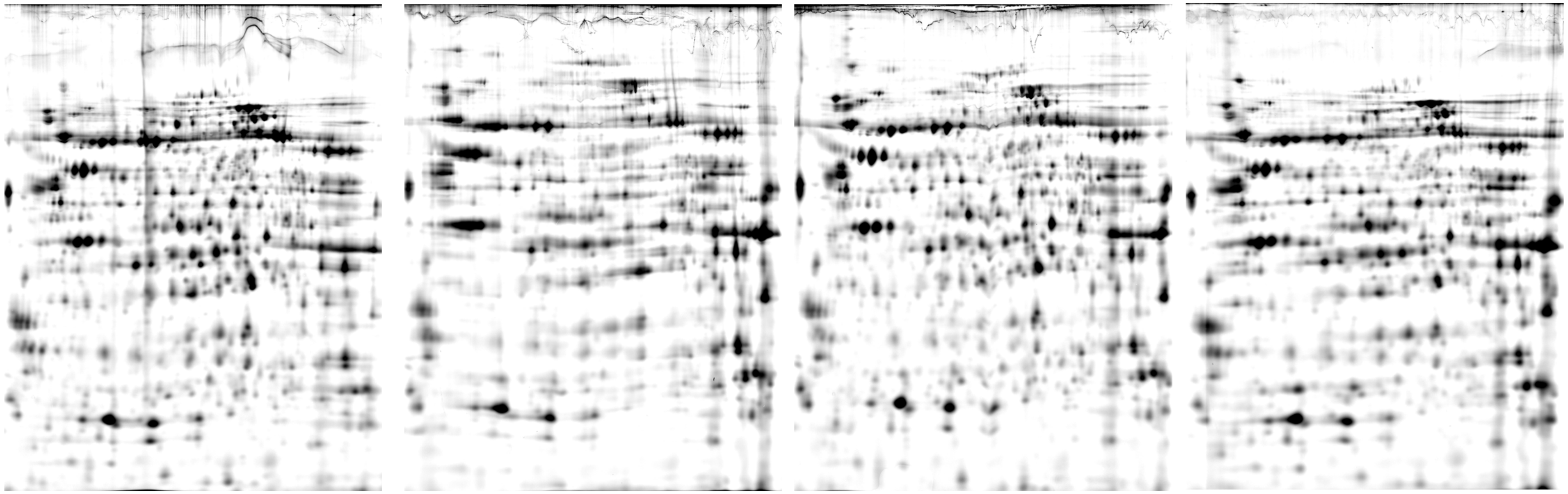

0,2µg dimethoate group:

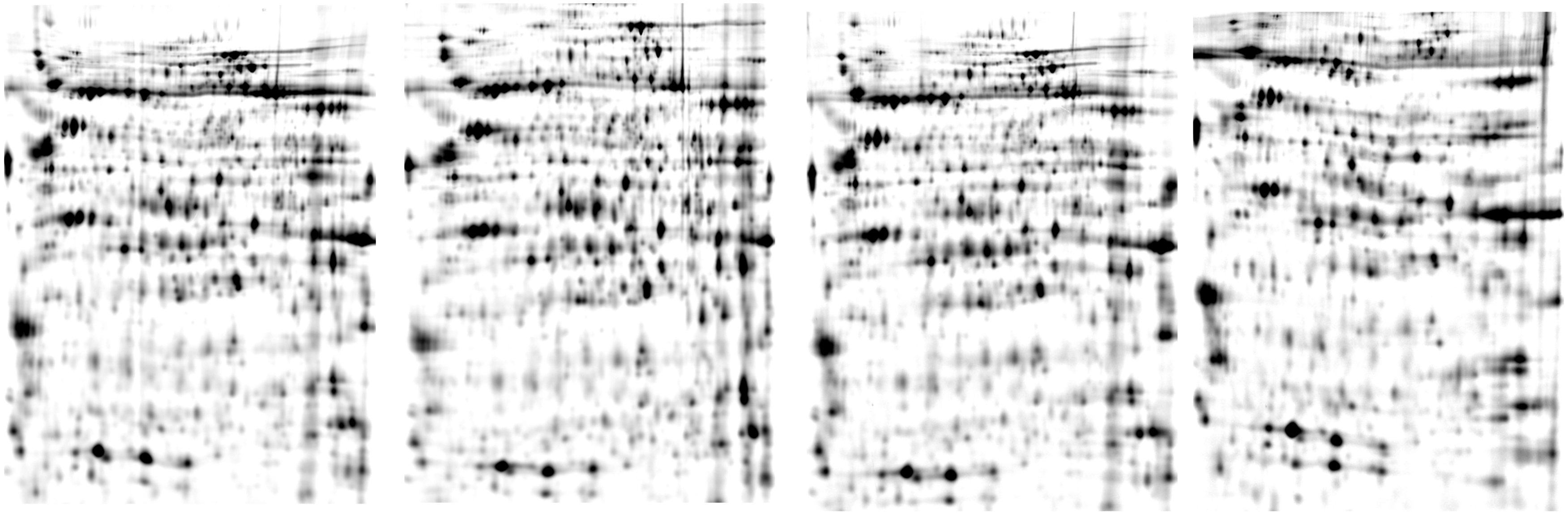

Fenoxycarb group:

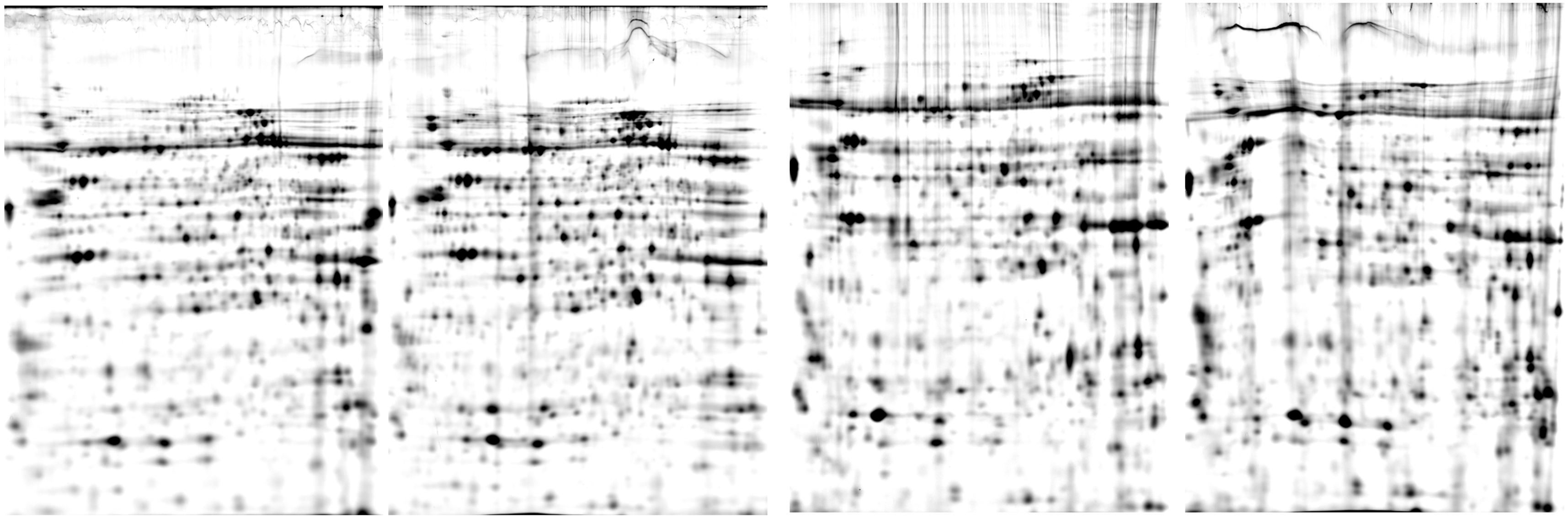

Flupyradifurone group:

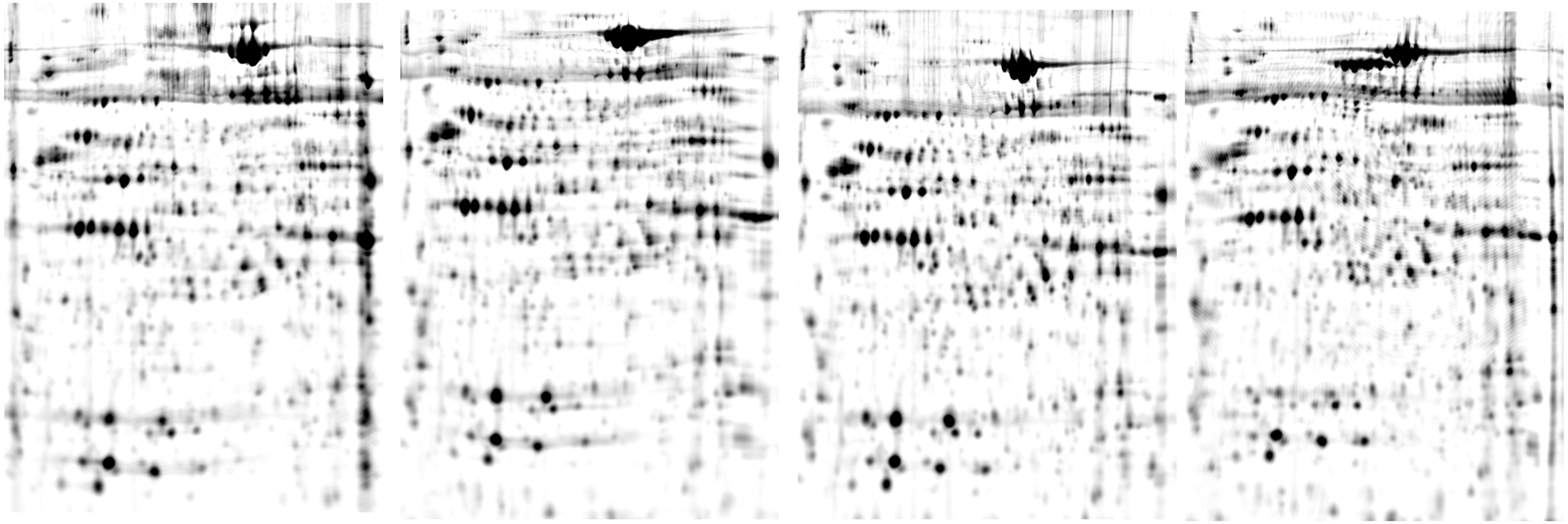

Supplement: Supplementary file 1 [file insects-14-00077-s001.zip › S3-replica Gel images.pdf]
